# Immobilization of Peroxidase onto Magnetite Modified Polyaniline

**DOI:** 10.1100/2012/716374

**Published:** 2012-03-12

**Authors:** Eduardo Fernandes Barbosa, Fernando Javier Molina, Flavio Marques Lopes, Pedro Antonio García-Ruíz, Samantha Salomão Caramori, Kátia Flávia Fernandes

**Affiliations:** ^1^Laboratório de Química de Proteínas, Instituto de Ciências Biológicas, Universidade Federal de Goiás, Codigo Postal 131, 74001-970 Goiânia, GO, Brazil; ^2^Grupo de Química de Carboydratos y Biotecnología de Alimentos, Departamento de Química Orgánica, Universidad de Murcia, Campus Espinardo, 34100 Murcia, Spain; ^3^Unidade Universitária de Ciências Exatas e Tecnológicas, Campus Dr. Henrique Santillo, Universidade Estadual de Goiás, Rodovia BR 153 Km 98, Codigo Postal 459, 75132-903 Anápolis, GO, Brazil

## Abstract

The present study describes the immobilization of horseradish peroxidase (HRP) on magnetite-modified polyaniline (PANImG) activated with glutaraldehyde. After the optimization of the methodology, the immobilization of HRP on PANImG produced the same yield (25%) obtained for PANIG with an efficiency of 100% (active protein). The optimum pH for immobilization was displaced by the effect of the partition of protons produced in the microenvironment by the magnetite. The tests of repeated use have shown that PANImG-HRP can be used for 13 cycles with maintenance of 50% of the initial activity.

## 1. Introduction

In the last decade, several works have been conducted with polyaniline (PANI) as the matrix for immobilization of horseradish peroxidase (HRP) and its application in the development of biosensors based on the conductive properties of the polymer support [1–6]. In its chemically synthesized form, PANI also has been studied as support for immobilization of several other enzymes [7–14]. Our experience with this support for immobilization of HRP include the relationship between the oxidation state of PANI and the amount of protein retained [7, 8], the acquired stability at extremes of pH, during long-time storage, under organic environment exposition, and during repeated use [8]. These studies culminated in the development of a highly stable device for “in flow” spectrophotometric determination of the hydrogen peroxide [15]. Considering PANI as support for enzyme immobilization and its use for the development of a batch reactor, the separation from reaction medium requires additional procedures such as centrifugation, filtration, or decantation, which are time and energy consuming. Attempts to overcome these problems include synthesis of composites of PANI, such as polyethylene terephthalate-PANI [16] and PVA-PANI [17] which can be manually removed from the reaction medium. In this case, the capacity of retention was drastically reduced as a consequence of reduction in the area for immobilization, similar to what occurs with electrochemically synthesized PANI.

Magnetization appears as a possible solution for several materials that are difficult to be removed from the reaction medium [18, 19]. In the present study, we present a magnetite-modified PANI, which assembles the high capacity of immobilization of the chemically synthesized polymer and its easy removal from the reaction medium by application of an external magnetic field in the bottom of the reaction flask. The magnetite-modified PANI was chemically and morphologically characterized, used for HRP immobilization, and the enzyme performance compared to that immobilized onto PANI. 

## 2. Methods

### 2.1. Polymer Synthesis, Magnetization, and Activation

Polyaniline (PANI) was synthesised as described previously [2] using ammonium persulphate as oxidising agent. The resulting green/black powder was submitted to magnetization followed by its activation by treating it with a solution of glutaraldehyde (PANImG). The magnetization consisted on the slowly addition of 10 mL of a solution of FeSO_4_ (121 mg mL^−1^) and FeCl_3_ (300 mg mL^−1^) to 100 mL of an aqueous suspension of PANI (20 mg mL^−1^). The mixture was alkalized to pH 12 by adding NH_4_OH 28% (v/v) and then it was heated to 80°C for 10 min (PANIm). The treatment with glutaraldehyde consisted on the incubation of PANIm in the solution 2.5% (v/v) under reflux for 2 h to produce glutaraldehyde-modified polyaniline (PANImG). The powdered PANImG was washed thoroughly with 0.1 mol L^−1^ phosphate buffer (pH 6.0), dried under vacuum, and stored at room temperature until further immobilization experiments.

### 2.2. Polymer Characterization

The polymer PANImG was characterized. PANIG and PANImG as KBr pellets were characterized by FT-IR analysis using a Perkin Elmer Spectrometer. Thermal gravimetric (TG) analyses were conducted in a Thermal analyzer (TA) Instruments (2920 MDSC V2.5F) with temperature ranging from 0 to 800°C with increasing of 10°C min^−1^. The scanning electron microscopy (SEM) analysis was conducted in a JEOL-6100 electronic microscope operated in 15 kV. The atomic force microscopy (AFM) analysis was performed in constant force contact mode using an SPM-9600 equipment (Shimadzu).

### 2.3. Optimization of Ph and Temperature of Immobilization

The optima time and pH for immobilization of peroxidase were tested by dissolving 10 *μ*g of HRP in 0.1 mol L^−1^ acetate buffer, pH 3.6–5.0, or in 0.1 mol L^−1^ phosphate buffer, pH 6.0–8.0, and 0.1 mol L^−1^ Tris/HCl, pH 9.0. These solutions of HRP (1.0 mL) were added to 5.0 mg of PANImG and the mixtures were gently stirred for 1 h at 4°C or room temperature. The solids were washed with the same buffer to remove unreacted enzyme and then tested for verifying the enzyme activity.

### 2.4. Assay of Immobilized Enzyme

Typical tests for free enzyme were performed by adding 500 *μ*L H_2_O_2_ (5.0 mmol L^−1^) to 2.5 mL of 0.1 mol L^−1^ phosphate buffer, pH 6.0, containing 0.013 mol L^−1^ pyrogallol and 1.0 *μ*g (16.2 U) free HRP. The reaction proceeded and after 1 min, the product was monitored at 420 nm in an Ultrospec 2000 spectrophotometer (Pharmacia). The tests for the immobilized enzyme were performed under identical conditions, except that the reaction was maintained under stirring and interrupted by separation of the HRP immobilized from the reaction mixture by using a magnet under the flask. The supernatants were transferred to a cuvette followed by spectrophotometrical readings. One unit of peroxidase activity (U) was defined as a change of 0.1 absorbance unit at 420 nm in 1 min. The values obtained in the blank reactions performed in the presence of either hydrogen peroxide or polymer (PANImG) were discounted from all the readings. All tests were performed in triplicate. The results were presented as average and standard deviation.

### 2.5. Repeated Use Tests

The tests of repeated use were performed as follows, with two concentrations of H_2_O_2_ (2.5 and 5.0 mmol L^−1^): 5.0 mg of PANImG-HRP were used in a reaction as described in the Section 2.4, then removed with aid of a magnet, washed three times with 0.1 mol L^−1^ phosphate buffer, pH 6.0, and then used in a new reaction.

## 3. Results and Discussion

### 3.1. Polymer Characterization

The chemical synthesis resulted in 88.3% yield. FT-IR analysis showed the characteristic bands of polyaniline and those related to modifications introduced by treatment with glutaraldehyde and magnetization (Table 1).

**Figure 1:**
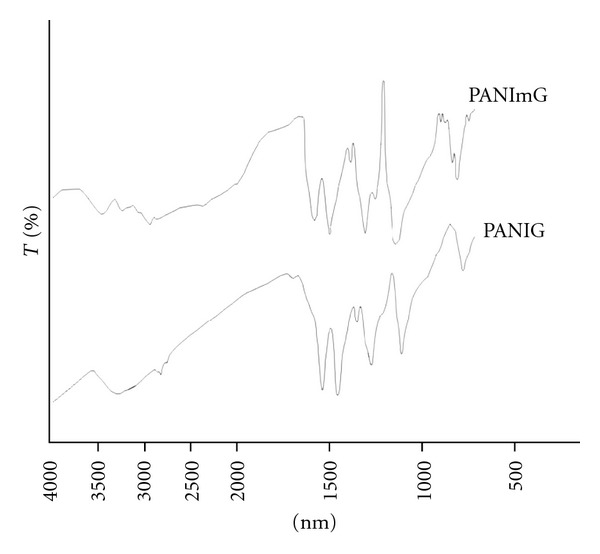
FT-IR spectra of PANIG and PANImG.

The main assignments of PANIG compared to PANI are the band at 2740 cm^−1^ and the band at 1720 cm^−1^ corresponding to aldehyde hydroxyl. After the magnetization, the band at 1720 cm^−1^ disappeared in PANImG, probably due to the recovering of the magnetite (Figure 1). The presence of magnetite can be confirmed by the band at 830 and 584 cm^−1^ due to the angular deformation and the hydroxyl groups from polymer surface due to the magnetite and due to stretching and torsional vibrations of magnetite Fe–O bands [20–22].

The decomposition curve characteristic of PANI has three stages of weight loss. The first step (50–120°C) indicates the loss of water in the matrix of the polymer; the second step of weight loss may be attributed to loss of acid dopand or volatile elements bounded to the polyaniline chain [21]. The third decomposition step occurs between 360 and 520°C, in which the mass loss may be a consequence of oxidative degradation of the polymer in the air. This stage may extend until 800°C, with complete degradation of carbonic backbone. The pattern of weight loss of magnetite modified PANIG is similar to unmodified PANI. Nevertheless, the remaining residue of PANImG is around 40% of the initial mass. This is due to the presence of magnetite, a compound that is not degraded in this range of temperature, whose metallic residue remains as ashes [23]. The increased thermal stability in magnetized polyaniline was also observed in other studies [22, 23].

**Figure 2:**
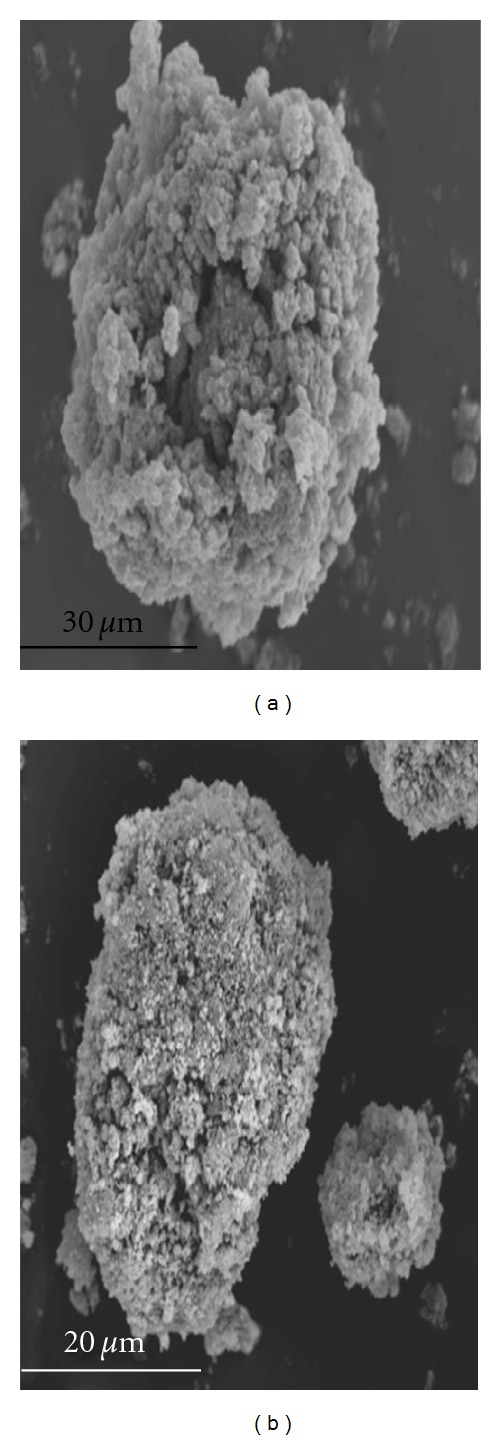
SEM micrographs of PANIG (a) and PANImG (b).

As can be seen at Figure 2(a), the polymer PANI shows a sponge-like structure, with a very large surface for enzyme immobilization. This structure also explains several properties observed in the different systems where polyaniline acts as support for enzyme immobilization. The cavities provide a microenvironment that protects the immobilized enzyme from denaturation. The magnetization (PANImG) resulted in a change in the texture of the surface of polyaniline (Figure 2(b)), which became rougher, but apparently without loses in its superficial area and consequently in its capacity for retention of proteins.

### 3.2. Parameters of Immobilization

**Figure 3:**
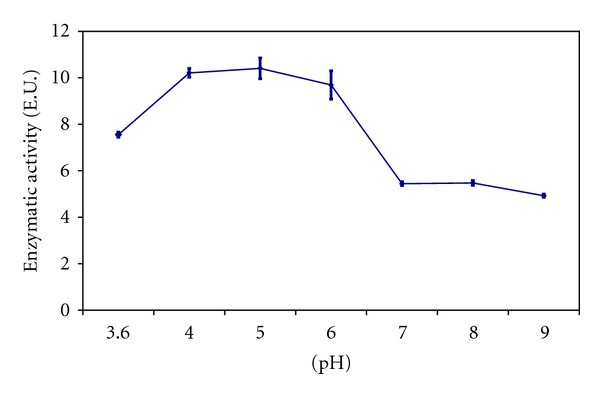
Effect of pH on immobilization of HRP in 5 mg of PANImG. Buffers used: 0.1 mol L^−1^ sodium acetate (pH 3.6–5.0); sodium phosphate 0.1 mol L^−1^ (pH 6.0–9.0).

Several conditions were tested to optimize the enzyme immobilization on the magnetite modified PANI.  Figure 3 shows the effect of solutions with different pH on the enzyme immobilization. The best results were obtained in the range between 4.0 and 6.0 for PANImG. Using PANIG, the best pH for the HRP immobilization was observed in the range among 6.0 and 8.0, with best performance at pH 6.0 [7]. The displacement observed in the pH of immobilization resulted from changes in the microenvironment produced by the magnetite. As reported by Bénézeth et al. [24], amino groups such as those presented in polyaniline induce changes in the behavior of the magnetite in acid pH. In this range of pH, when magnetite is closely bonded or deposited on a support with available amino groups, magnetite acts as a receptor of protons as described by

(1) $$\equiv {\text{FeOH}}^{-0.5}\;+\;{{\text{H}}^{+}}_{\left(\text{aq}\right)}⟷\;\equiv {{\text{FeOH}}_{2}}^{+0.5}.$$

The consequence of the capacity of removing protons from the microenvironment is an increase in the pH value in the neighborhood of the PANImG, while the pH in the bulk remains acid. In this case, the groups involved in the bonding of the HRP are the same groups that react with PANIG, since the pH values in the microenvironment of PANIG and PANImG are the same. This hypothesis is supported when comparing the efficiency of immobilization of HRP in both PANIG and PANImG.

The efficiency of immobilization was the same at 4°C and at room temperature. The amounts of enzyme and protein bonded to magnetite modified PANI after optimizing the immobilization conditions were the same as those obtained with PANIG. The best result shows that 25.2% of the enzyme offered and 24% of the protein available for immobilization process were immobilized. This indicates that 100% of the immobilized protein retained its native activity. Moreover, this result reinforces the same chemical behavior in the immobilization reaction, instead of the pH displacement.

### 3.3. Repeated Use Tests

**Figure 4:**
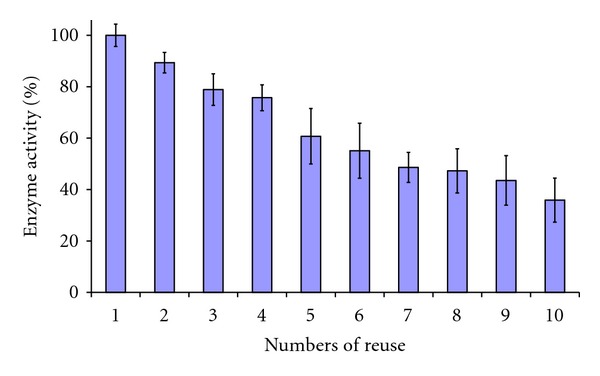
Evaluation of peroxidase activity maintenance after repeated use of HRP-PANImG. Assay condition: 0.1 mol L^−1^ sodium phosphate buffer pH 6.0 and 5.0 mmol L^−1^ H_2_O_2_.

The tests of repeated use of PANIG-HRP and PANImG-HRP revealed performances quite different. PANImG-HRP presented performance inferior to PANIG-HRP in repeated use, indicating that magnetite could play an important role in the stability of the HRP. PANImG-HRP showed great losses in activity in each cycle of using and washing, retaining only 50% of its initial activity at the 9th cycle of repeated use (Figure 4).

Similar result was found by Hong et al. [25], working with magnetic nanogels polymers, which were first magnetized and then activated.

It is possible that magnetite in the surface of PANImG acts as a charged group for electrostatic interactions with HRP and then, the enzyme molecules weakly bonded were leached at each cycle of washing with phosphate buffer 0.1 mol L^−1^.

**Figure 5:**
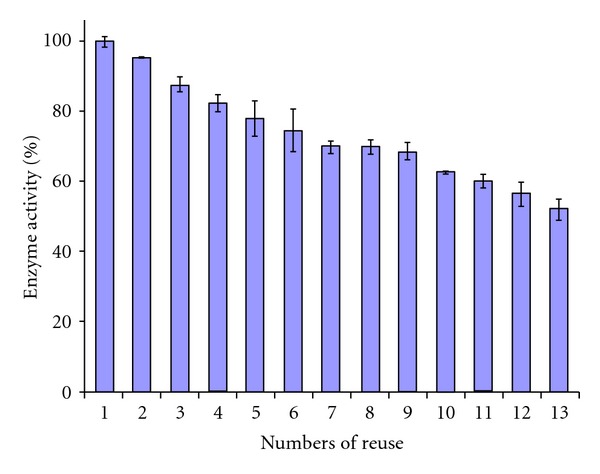
Evaluation of peroxidade activity maintenance after repeated use of HRP-PANImG with H_2_O_2_ in lower concentration (2.5 mmol L^−1^).

The irreversible inhibition of HRP by high concentration of hydrogen peroxide is a well-known characteristic of this enzyme [26, 27]. Otherwise, some works reported that magnetite presents oxyreductive properties. In this work, the PANImG was able to oxidize catechol in absence of HRP (the values were discounted as blank values). Hence, the interaction between hydrogen peroxide and magnetite could result in early HRP inactivation. To verify this hypothesis, tests of repeated use were conducted in concentrations of hydrogen peroxide 50% lower (2.5 mmol L^−1^) than used in normal assay (5.0 mmol L^−1^). The results are presented in Figure 5.

As can be seen, reduced hydrogen peroxide concentration enhanced stability for PANImG-HRP, being the last system more stable, retaining 50% of the initial activity after 13 cycles of repeated use. This result indicates that an interaction between magnetite and hydrogen peroxide may be responsible for HRP inactivation.

## 4. Conclusion

This study shows that magnetite-modified PANI is suitable for immobilization, conferring to the system the advantage of being easily removed from the reaction medium by applying a magnetic field.

The immobilization of HRP on magnetite-modified PANI had the same efficiency obtained with PANI without magnetization. The magnetization and activation does not interfere in the amounts of enzyme immobilized, but it does interfere in the stability of the immobilized enzyme. It occurs probably due to the interactions between the magnetite and the hydrogen peroxide. The present study showed that working with hydrogen peroxide in concentration of 2.5 mmol L^−1^ resulted in an increase of stability for HRP, which was used in 13 cycles of reaction.

## Figures and Tables

**Table 1:** Comparative FT-IR bands of PANI, PANIG, and PANImG.

Polymer	Assignments	Band
PANI*	NH from benzoid ring	1500 cm^−1^
	NH from quinoid ring	1600 cm^−1^
	doping grade	1100 cm^−1^

PANIG**	aldehyde hydroxyl	1720 cm^−1^
	assimetric stretching of CH_2_	2740 cm^−1^

PANImG	angular deformation	830 cm^−1^
	superficial hydroxyl group	584 cm^−1^

*Data from Fernandes et al. [7].

**Data from Azevedo et al. [20].
